# Supplementary material for: Reversible Myc hypomorphism identifies a key Myc-dependency in early cancer evolution
Source: Nat Commun. 2022 Nov 9;13:6782. doi: 10.1038/s41467-022-34079-x (PMC9646778; doi:10.1038/s41467-022-34079-x)

SUPPLEMENTARY FIGURE LEGENDS

Supplementary Figure 1: Design of the reversibly switchable *Myc*^{TRE} hypomorph allele

a. Schematic of wild type endogenous *Myc* gene, the targeting vector and the resultant *Myc* targeted allele (not to scale) with *TRE* insertion in intron 2. "*3" refers to silent mutations incorporated into exon 3 of the targeted *Myc* allele to distinguish it from that of the endogenous wild type Myc.

b. Schematic of mechanism of action of reversible tTS^{Kid}-regulated hypomorphism of the endogenous the *Myc* gene by tetracycline.

Sodir *et al.* Supplementary Figure 1:
Design of the reversibly switchable *Myc*^{TRE} hypomorph allele

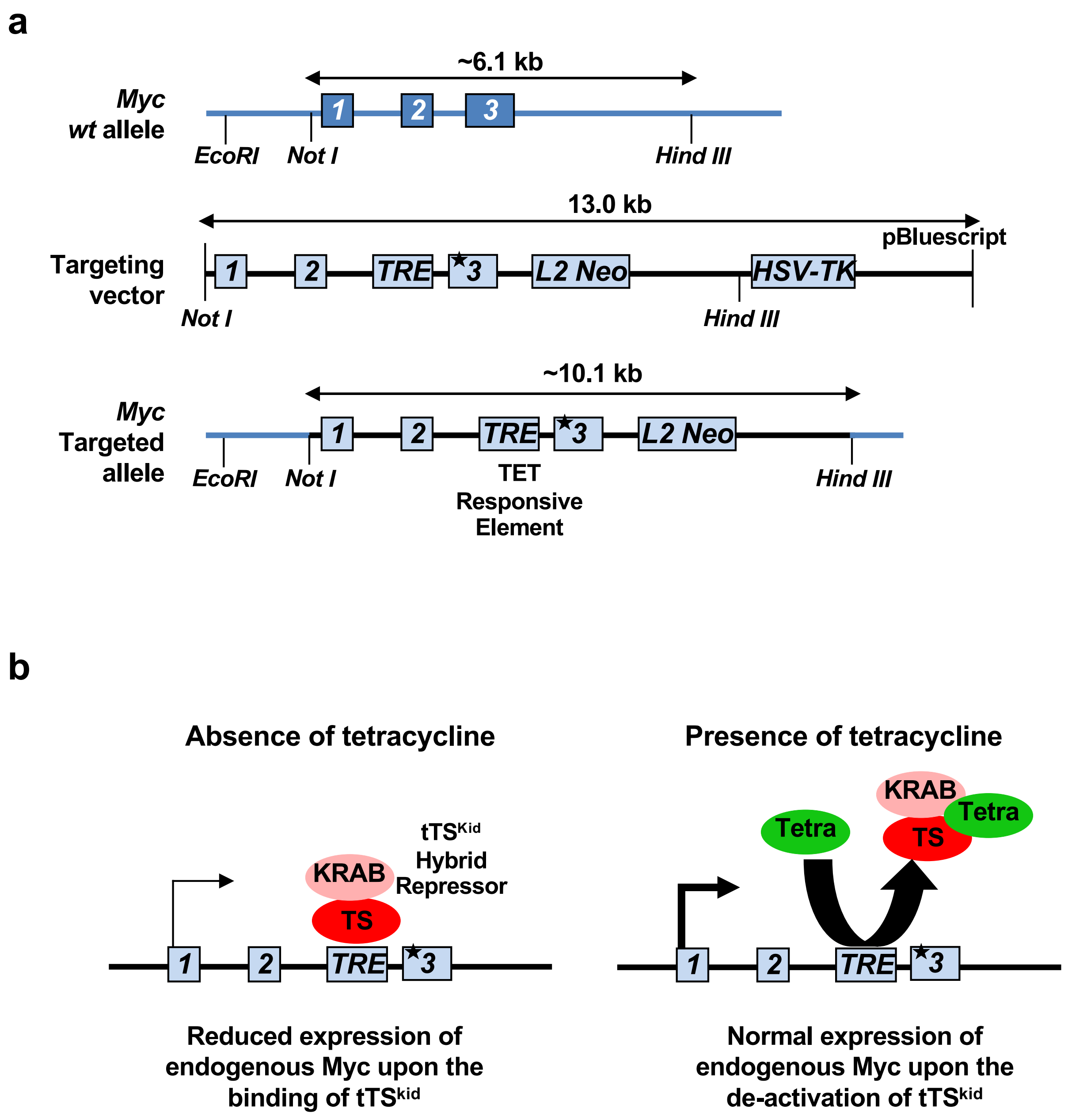

Supplementary Figure 2: *TRE* insertion insertion into the endogenous c-*myc* gene 2nd intron does not measurably impact endogenous Myc expression level or kinetics of serum regulation

a. Scheme for analysis of mitogenic induction of Myc. Mouse adult lung fibroblasts (MALFs) were isolated from $Myc^{TRE/TRE}$ or *wt* adult mice (with n=2 per group) and maintained in normal medium (DMEM with 10% FBS). Prior to mitogen induction, MALFS were cultured in 0.1% FBS for 72 hrs to induce quiescence and then serum-stimulated (20% FBS) for 0, 2, 6 and 12 hrs.

b. Kinetics of serum induction of Myc in quiescent control (*wt*) versus $Myc^{TRE/TRE}$ MALFS, showing identical signature transient Myc induction. Cells were collected and lysed in RIPA buffer and whole protein lysates (5 µg) were subjected to Western blot analysis for Myc. Two biologically independent MALFs per group were tested with one shown for simplicity.

c. Left: Comparison of Myc levels in asynchronous log-phase $Myc^{TRE/TRE}$;$tTS^{Kid/–}$ MALFs growing in either the absence (hypomorphed) or presence (non-hypomorphed) of 1mg/ml of doxycyline

Right: ImageJ quantification of relative Myc protein levels (normalized to β-actin loading control) in asynchronous growing $Myc^{TRE/TRE}$;$tTS^{Kid/–}$ MALFS in the absence or presence of doxycycline. Two biologically independent MALFs per group were tested with one shown for simplicity.

Source data are provided as a Source Data file.

Sodir *et al.* Supplementary Figure 2:
***TRE* insertion insertion into the endogenous c-*myc* gene 2nd intron does not measurably impact endogenous Myc expression level or kinetics of serum regulation**

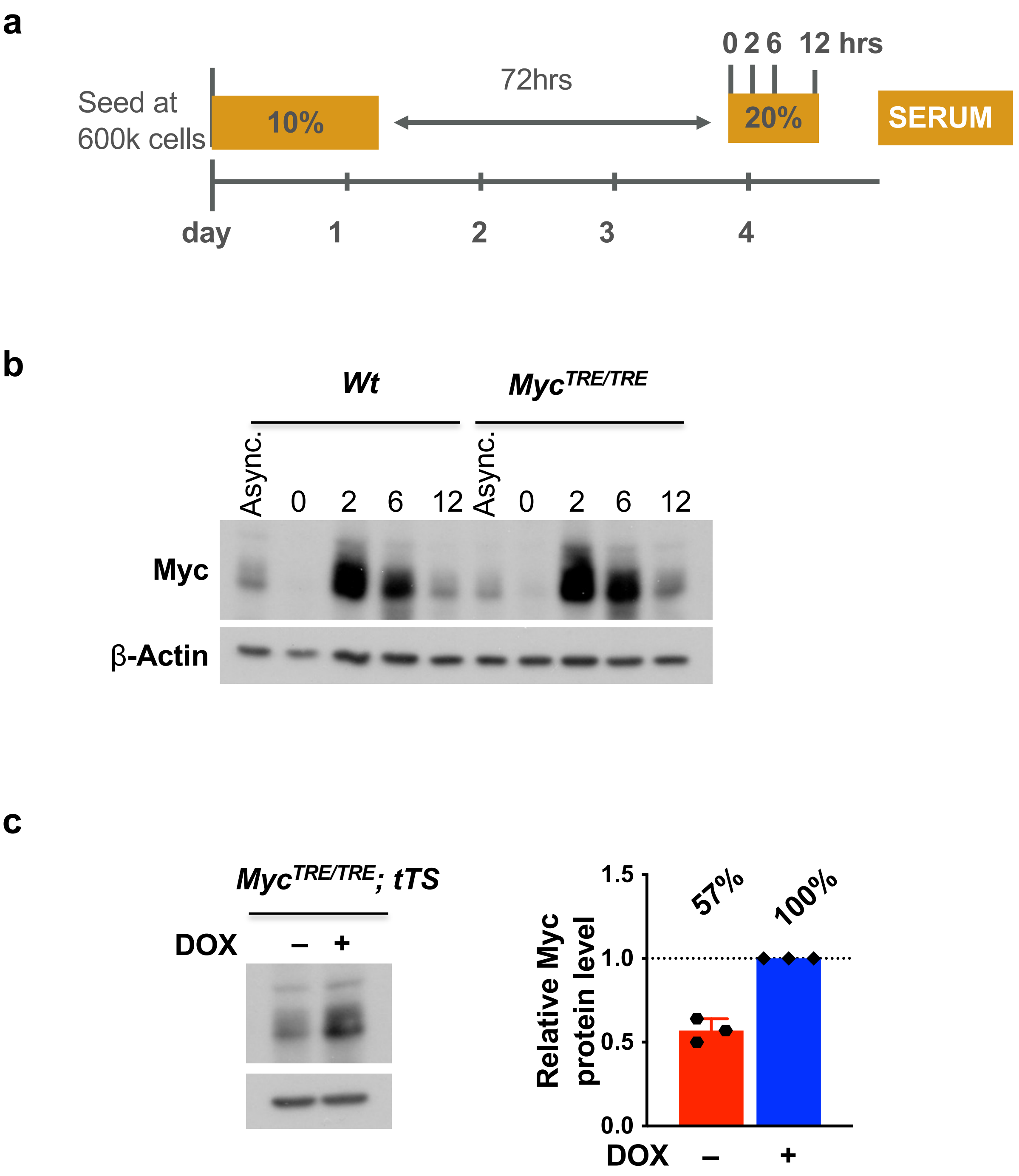

Supplementary Figure 3: Hypomorphism of endogenous Myc imposed in proliferative adult mouse tissues is rapidly and fully reversible, and has no significant deleterious impact on proliferative tissues

a. Withdrawal of tetracycline from *MR* mice induces reversible Myc hypomorphism. Quantitative RT-PCR analysis of Myc mRNA expression levels in bone marrow, spleen, thymus, and small intestine from adult MR mice without tetracycline (i.e. Myc hypomorphed) for 0 (blue), 2, and 4 weeks (red), or without tetracycline for 4 weeks and then followed by restoration of tetracycline for 1 week (black). *Tbp* mRNA serves as a control gene. Results depict *Myc* expression mean + SD. The unpaired t-test with Welch's and two-tailed analysis correction was used to analyze data. In Bone marrow, n= 4 mice on tetracycline and n= 5, 3, and 4 mice with p=0.0177, 0.0191, and 0.7505 for 2, 4, 4/+1 weeks post tetracycline withdrawal/restoration, respectively and relative to the vehicle control. In Spleen, n= 6 mice on tetracycline and n=5, 3, and 4 mice with p=0.0002, 0.0056 and 0.8275 for 2, 4, 4/+1 weeks post tetracycline withdrawal/restoration, respectively and relative to the vehicle control. For thymus, n=6 on tetracyline and n= 4, 3, and 4 mice with p=0.0109, 0.0008, and 0.6511 for 2, 4, 4/+1 weeks post tetracycline withdrawal/restoration, respectively and relative to the vehicle control. For small intestine n= 6 mice on tetracycline and n= 5, 3 and 4 mice with p=0.0033, 0.0089, 0.1786 for 2, 4, 4/+1 weeks post tetracycline withdrawal/restoration, respectively and relative to the vehicle control.

b. Imposition of Myc hypomorphism for 4 weeks has no evident impact on architecture of proliferative *MR* mouse tissues. Representative H&E-stained sections of bone marrow, spleen, thymus and small intestine from, *MR* mice tetracycline treated throughout (non-hypomorphed control) versus 4-week without tetracycline (Myc hypomorphed).

c. Imposition of Myc hypomorphism for 4 weeks has no significant impact on *MR* mouse haematopoiesis. Peripheral blood was collected from tetracycline treated (blue) or tetracycline deprived (red) MR mice. Data depict mean +SD of blood counts for leukocytes (left), erythrocytes (middle) and platelets (right) relative to tetracycline treated mice. The unpaired t-test with Welch's

correction and two-tailed analysis was used to analyze data. For leukocytes, n= 4 mice on tetracycline and n= 4 and 10 mice with p= 0.8014 and 0.6572 for 2, 4 weeks post tetracycline withdrawal, respectively and relative to the vehicle control. For erythrocytes, n= 4 mice on tetracycline and n= 4 and 11 mice with p= 0.6206 and 0.8201 for 2, 4 weeks post tetracycline withdrawal, respectively and relative to the vehicle control. For Platelets, n= 4 mice on tetracycline and n=3 and 11 mice with p=0.8911 and 0.3467 for 2, 4 weeks post tetracycline withdrawal, respectively and relative to the vehicle control.

*p < 0.05, **p < 0.01, ***p < 0.001, ns = non-significant, SD = Standard deviation.

Source data are provided as a Source Data file.

Sodir *et al.* Supplementary Figure 3:
Hypomorphism of endogenous Myc imposed in proliferative adult mouse tissues is rapidly and fully reversible, and has no significant deleterious impact on proliferative tissues

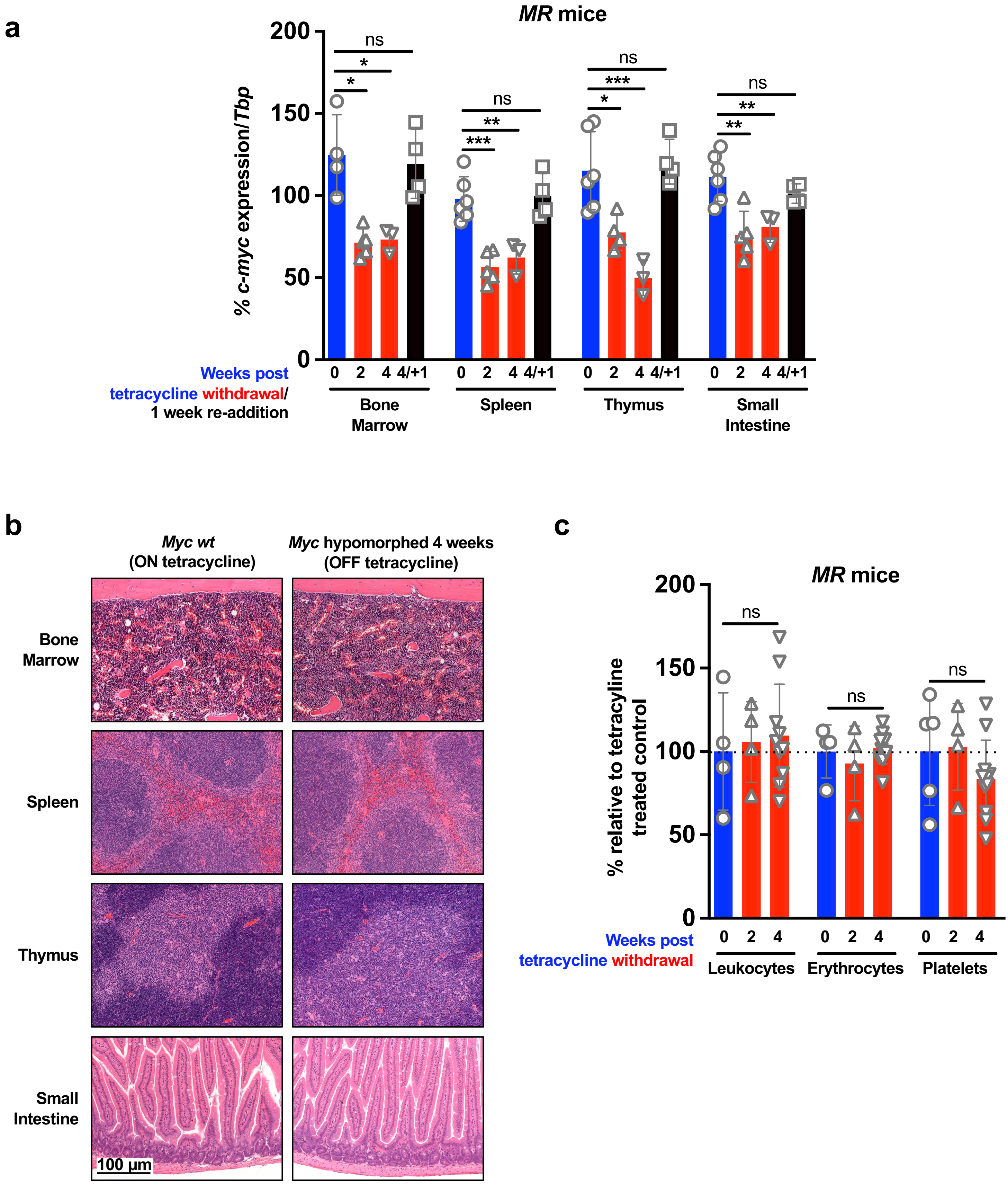

Supplementary Figure 4: Induction of Myc hypomorphism in neonatal *MR* mouse lung and pancreas

a. Schematic representation of study to assess impact of post-partum imposition of Myc hypomorphism on subsequent neonatal lung and pancreas development. $M^{TRE/TRE}$ females were mated with $M^{TRE/TRE};R^{+/-}$ males and pregnant females were maintained from conception on tetracycline until birth to maintain normal endogenous Myc levels. From birth (day 0) Myc was hypomorphed by cessation of tetracycline administration and neonatal mice were then euthanized at 8 or 14 days of age for analysis. Control neonates maintained on tetracycline were euthanized at the same time points.

b. Tetracycline treatment regulates Myc hypomorphism in neonatal *MR* lung and pancreas. Quantitative RT-PCR analysis of *Myc* mRNA isolated from lungs (left) and pancreas (right) of 8 and 14 day-old control non-hypomorphed $M^{TRE/TRE};R^{+/-}$ (blue) versus $M^{TRE/TRE};R^{+/-}$ neonates hypomorphed from birth (red). Results depict mean + SD. *Tbp* was used as a control "housekeeping" gene. The unpaired t-test with Welch's correction and two-tailed analysis was used to analyze Taqman expression data. For lung, n=3 for non-hypomorphed control and n=5 for hypomorphed mice at 8 d with p=0.0002 and n=4 for non-hypomorphed control and n=3 for hypomorphed mice at 14 d with p=0.0007. For pancreas, n= 4 for both non-hypomorphed control and hypomorphed mice at 8 d with p=0.0346 and n=3 for both non-hypomorphed control and hypomorphed mice at 14 d with p=0.0053.

*p < 0.05, **p < 0.01, ***p < 0.001. SD = standard deviation.

Source data are provided as a Source Data file.

Sodir *et al.* Supplementary Figure 4:
Induction of Myc hypomorphism in neonatal *MR* mouse lung and pancreas

a

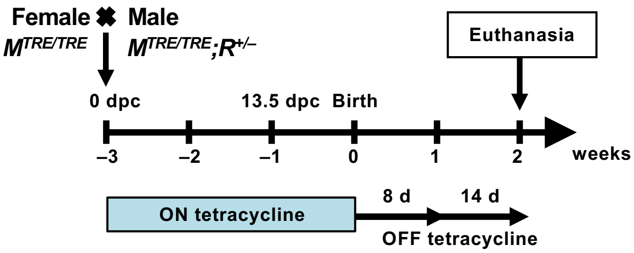

b

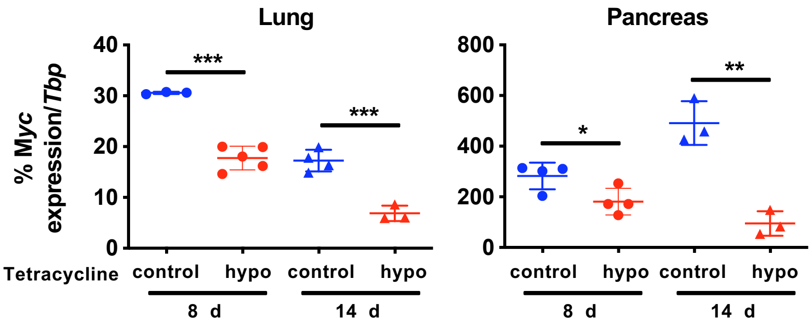

Supplementary Figure 5: Absence of p53 promotes adventitious genetic mechanisms that break the *MR* mouse model

Occasional p53-deficient lung and pancreas *MR* tumours sporadically appear to escape Myc hypomorphism. However, in most instances escape is due to silencing expression of the tTs^{KID} repressor that induces Myc hypomorphism and/or by amplifying Myc expression so that Myc is no longer constrained to hypomorphic levels.

a. Left: Top panels show RNAscope staining for tTS^{Kid} repressor expression in a representative escapee lung tumour. Whereas tTS^{Kid} is broadly expressed by lung tumour cells that develop in non-hypomorphed $MRKP^{fl}$ mice (left) it is absent from an escapee $MRKP^{fl}$ mouse lung tumour arising in a hypomorphed mouse (right). Bottom panels show representative images of Myc IHC staining in lung tumours arising in non-hypomorphed mice (left). Myc is sometimes elevated in such tumours (~18%) but not all. By comparison, Myc is dramatically elevated in a high proportion (60%) of large escapee lung tumour (right) that arise in “hypomorphed” (off tetracycline) $MRKP^{fl}$ animals.

Right: quantification of incidence of escape mechanisms in individual “escapee” lung tumours, showing (top) expression of the tTS^{Kid} repressor (RNA ISH) and (bottom) elevated levels of Myc (Myc IHC positive cells >25%) relative to control non-hypomorphed (ON tetracycline). Escapee tumours were isolated from hypomorphed $MRKP^{fl}$ mice at 14 weeks post Adv-Cre infection. Results represent percentage of lesions (n) per lung section per mouse, mean ± SD. The unpaired t-test with Welch’s correction and two-tailed analysis was used to analyze the data. For tTS expression, n=4 mice for both non-hypomorphed control and hypomorphed mice with p=0.0267. For Myc expression, n= 3 mice for non-hypomorphed control and n= 4 mice for hypomorphed mice with p= 0.0019.

The total number of lesions analyzed ranged from 17 to 71 per lung section per mouse.

b. Top left: representative RNAscope *in situ* hybridization analysis showing tTS^{Kid} expression in PDAC from non-hypomorphed (no tetracycline) *MRKPC* mice and its absence from an escapee PDAC tumour (+tetracycline). Boxes represent regions shown at higher magnification below.

Top right: quantitation of RNAscope analysis of *tTS*^{Kid} expression in PDACs from non-hypomorphed *MRKPC* mice (blue) versus "hypomorphed" PDAC escapees (red). The *tTS*^{Kid} score for each PDAC was calculated as follows: score 1 = <25% of PDAC cells express *tTS*^{Kid} RNA, score 2 = 25% to 50% of PDAC cells express *tTS*^{Kid}, score 3 = >50% to 75% of PDAC cells express *tTS*^{Kid}, score 4 = >75% of PDAC cells express *tTS*^{Kid}. The unpaired t-test with Welch's correction and two-tailed analysis was used to analyze the data. Mean ± SD are shown. For *tTS*^{Kid} expression, n= 6 independent PDAC tumors (4 mice) for non-hypomorphed control and n=5 independent PDAC tumors (4 mice) for hypomorphed mice with p< 0.0001. For Myc expression,

Bottom left: Myc IHC staining in representative examples of PDACs from non-hypomorphed *MRKPC* mice (left) versus hypomorphed PDAC escapee (right). Boxes indicate regions shown at higher magnification below.

Bottom right: quantitation of cells positive for Myc expression (IHC) in PDACs from non-hypomorphed *MRKPC* mice (blue) versus escapee PDACs from hypomorphed counterparts (red). The Myc score for each PDAC was calculated as follows: score 1 = <20% of PDAC cells express Myc, score 2 ≥ 20% of PDAC cells express Myc. The unpaired t-test with Welch's correction and two-tailed analysis was used to analyze the data. Mean ± SD are shown. For Myc expression, n= 7 independent PDAC tumors (5 mice) for non-hypomorphed control and n=5 independent PDAC tumors (4 mice) for hypomorphed mice with p= 0.0082.

*p < 0.05, **p < 0.01, ***p < 0.001, ****p < 0.0001. FOV = field of view. SD = standard deviation.

Source data are provided as a Source Data file.

Sodir *et al.* Supplementary Figure 5:
Absence of p53 promotes adventitious genetic mechanisms that break the *MR* mouse model

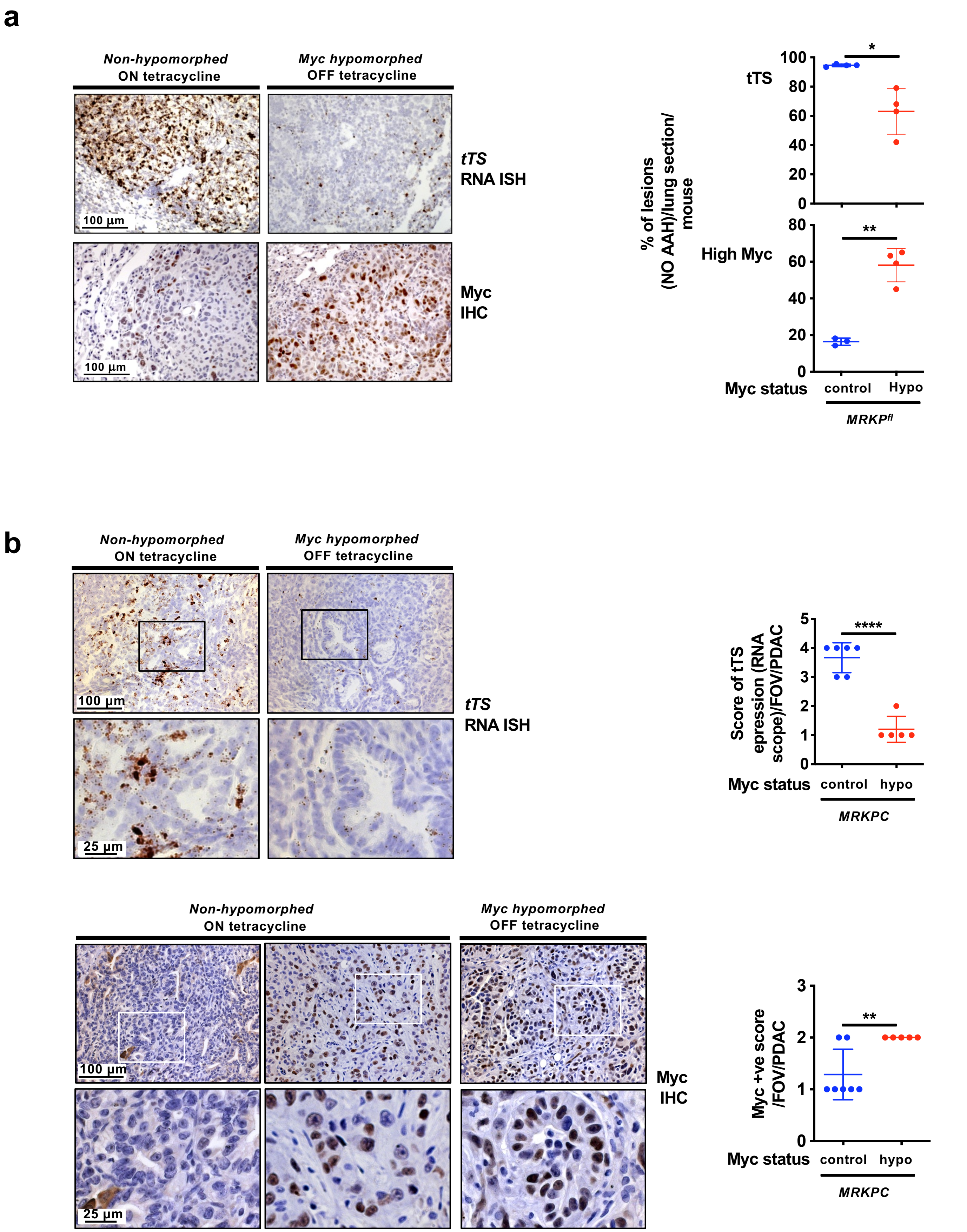

Supplementary Figure 6: Hypomorphic Myc levels that are insufficient to drive transition to pancreatic adenocarcinoma nonetheless retain capacity to induce most Myc target genes

Heat map of Myc target gene RNA expression in pancreas samples from 12 week-old $KCR26M^{MT2/+}$ (1 copy $MycER^{T2}$ – Myc at hypomorphic level) and $KCR26M^{MT2/MT2}$ (2 copies $MycER^{T2}$ – Myc at ~physiological level) mice. $MycER^{T2}$ was activated for 12 hours (Myc ON) or never activated (Myc OFF). Representative Myc target genes were chosen from HALLMARK_MYC_TARGETS_V1 (GSEA, M5926), HALLMARK_MYC_TARGETS_V2 (GSEA, M5928), DANG_MYC_TARGETS_UP (GSEA, M6506) and MYC_TARGETS [47] gene sets. The heat map shows fold change (FC) in target gene expression in, respectively, $KCR26M^{MT2/+}$ (Myc ON versus Myc OFF) and $KCR26M^{MT2/MT2}$ (Myc ON versus Myc OFF). Target genes are ranked based on their expression levels in $KCR26M^{MT2/\ MT2}$. n=4 mice per group. All RNA-seq data have been deposited in the ArrayExpress database at EMBL-EBI (www.ebi.ac.uk/arrayexpress) under accession number E-MTAB-10807.

Source data are provided as a Source Data file.

Sodir *et al.* Supplementary Figure 6:
Hypomorphic Myc levels that are insufficient to drive transition to pancreatic adenocarcinoma nonetheless retain capacity to induce many Myc target genes

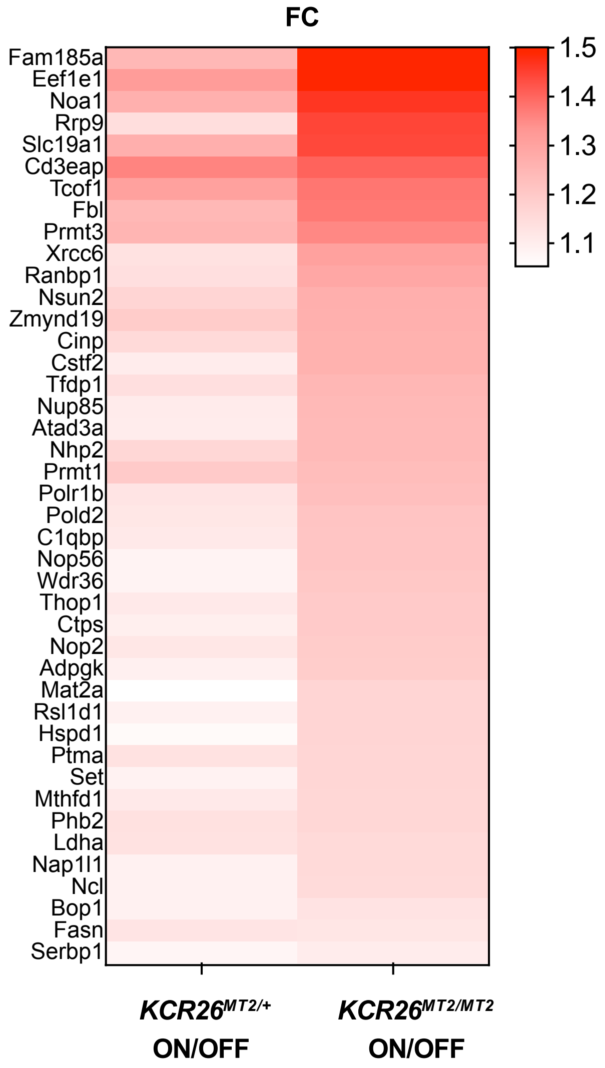

Supplementary Figure 7: Imposition of Myc hypomorphism from conception triggers *MR* embryo failure

a. Table of number, fate and corresponding genotypes of embryos/pups derived by crossing *M* (M^{TRE}/M^{TRE}) females with *MR* ($M^{TRE}/M^{TRE};R^{+/-}$) males and collected at 8.5, 9.5, 10.5, 12.5, and 13.5 *dpc*, and post-weaning.

b. Representative images of 10.5, 12.5 and 13.5 *dpc* littermate embryos derived by crossing *M* (M^{TRE}/M^{TRE}) females with *MR* ($M^{TRE}/M^{TRE};R^{+/-}$) males as described above (A) and maintained from conception in a Myc-hypomorphed state (i.e. absence of tetracycline).

c. Surviving embryos and corresponding genotypes of embryos/pups resulting from ♀*MR* ($M^{TRE}/M^{TRE};R^{+/-}$) X ♂*MR* (M^{TRE}/M^{TRE}) crosses. *MR* Females were hypomorphed by tetracycline withdrawal for 4 weeks before they were bred with M males while maintained without tetracycline for 6 weeks (–). No pups, whether M^{TRE}/M^{TRE} or $M^{TRE}/M^{TRE};R^{+/-}$, were born. Tetracycline was then re-administered, whereupon *MR* females became pregnant after about 4 weeks (–/+).

Sodir *et al.* Supplementary Figure 7:
Imposition of Myc hypomorphism from conception triggers *MR* embryo failure

a

Tetracycline	♀ $M^{TRE/TRE}$ X ♂ $M^{TRE/TRE}$;$R^{+/-}$			
		Progeny		
	Age (# litters)	$M^{TRE/TRE}$	M^{TRETRE};$R^{+/-}$	Resorbed embryos
+	Adult (6)	19	23	ND
–	Adult (5)	17	0	ND
	13.5 dpc (1)	3	0	5
	12.5 dpc (3)	9	6	5
	10.5 dpc (1)	5	5	0
	9.5 dpc (1)	5	3	0
	8.5 dpc (1)	6	6	0

b

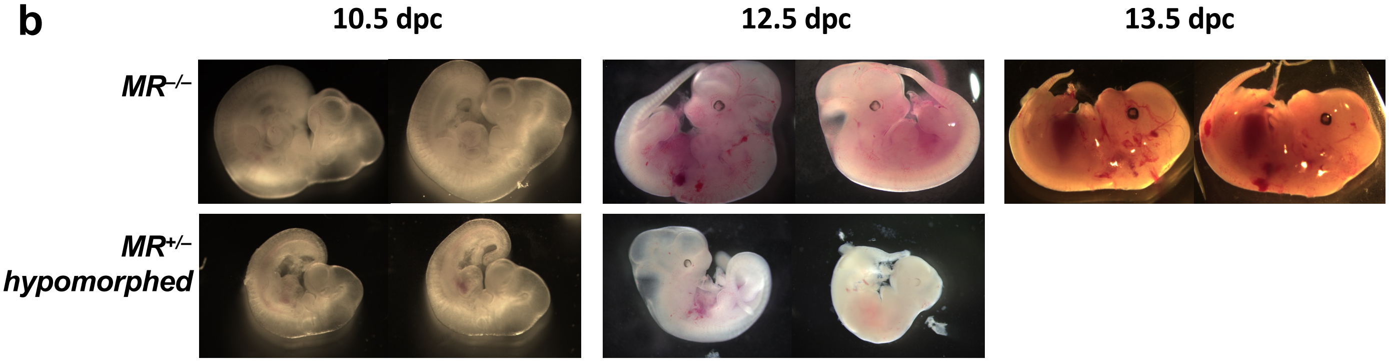

c

Tetracycline	♀ $M^{TRE/TRE}$;$R^{+/-}$ X ♂ $M^{TRE/TRE}$		
		Progeny	
	Age (# litters)	$M^{TRE/TRE}$	M^{TRETRE};$R^{+/-}$
–	Adult (0)	0	0
–/+	Adult (4)	14	15

Supplementary Figure 8: Post-partum imposition of Myc hypomorphism has no discernible impact on postnatal lung or pancreas architecture or proliferation, nor on overall neonatal growth

a. Top: Representative H&E-stained sections of lung and pancreas harvested from control versus neonatally hypomorphed at 8 and 14 days post-partum.

Middle: Representative Ki67 IHC-stained sections harvested from control versus neonatally hypomorphed at 8 and 14 days post-partum, as described above.

Bottom: Relevant quantification of IHC for Ki67 in sections of lung and pancreas harvested from control versus neonatally hypomorphed at 8 and 14 days post-partum. Results depict mean ± SD, n = 4 mice per treatment group. The unpaired t-test with Welch's correction and two-tailed analysis was used to analyze data. For lung, n=4 for both non-hypomorphed control and hypomorphed mice at 8 d and 14 d with p=0.7611 and p=0.6057, respectively. For pancreas, n=4 for both non-hypomorphed control and hypomorphed mice at 8 d and 14 d with p=0.9670 and p=0.4413, respectively.

b. Body weights of 8 and 14 day old non-hypomorphed control $M^{TRE/TR};R^{+/-}$ (blue) versus $M^{TRE/TRE};R^{+/-}$ neonatally hypomorphed from birth (red). Data depicts mean ± SD. The unpaired t-test with Welch's correction and two-tailed analysis was used to analyze data. n=16 for non-hypomorphed control and n= 15 for hypomorphed mice at 8 d with p=0.7590; n=6 for non-hypomorphed control and n= 12 for hypomorphed mice at 14 d with p=0.8950. ns = non-significant**.**

Source data are provided as a Source Data file.

Sodir *et al.* Supplementary Figure 8:

Post-partum imposition of Myc hypomorphism has no discernible impact on postnatal lung or pancreas architecture or proliferation, nor on overall neonatal growth

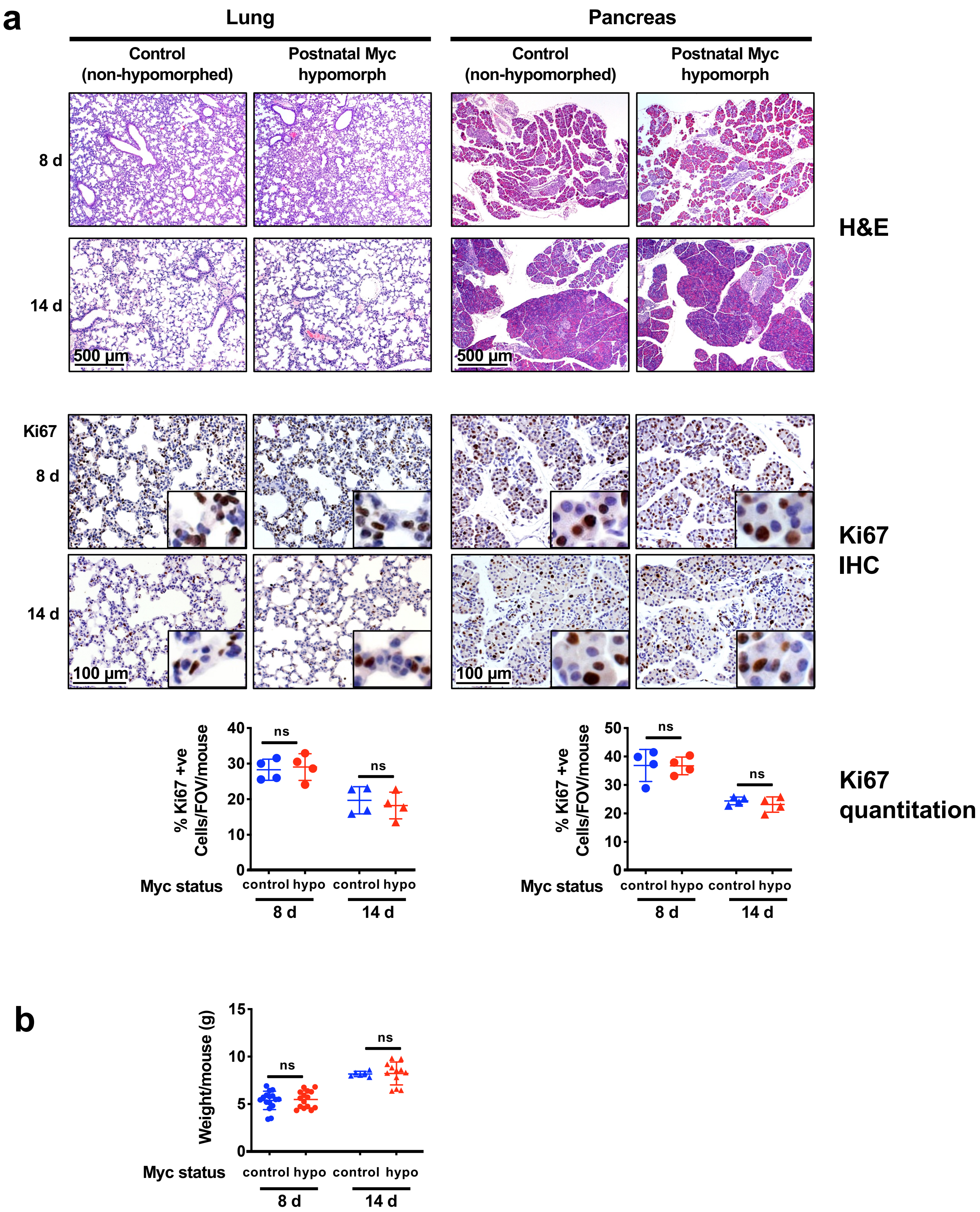

Supplementary Figure 9: Imposition of Myc hypomorphism immediately after 13.5 *dpc* has no discernible impact on subsequent development or neonatal growth

a. Schematic of study. Embryos from $M^{TRE/TRE}$ females crossed with $M^{TRE/TRE};R^{+/-}$ males were maintained on tetracycline (normal Myc levels) until 13.5 *dpc*. Tetracycline was then withdrawn to induce Myc hypomorphism and neonates euthanized 8 days later. Control embryos were maintained on tetracycline throughout.

b. Quantitative RT-PCR analysis of *Myc* mRNA isolated from lungs and pancreas of 8 day old control $M^{TRE/TRE};R^{+/-}$ mice maintained on tetracycline (non-hypomorphed - Blue) versus 8 day-old $M^{TRE/TRE};R^{+/-}$ neonates hypomorphed post 13.5 *dpc* (hypomorphed – Red). Results depict mean + SD. *Tbp* was used as a control gene. The unpaired t-test with Welch's correction and two-tailed analysis was used to analyze data. For lung, n=4 for non-hypomorphed control and n=4 hypomorphed mice with p=0.0149. For pancreas, n=3 for both non-hypomorphed control and hypomorphed mice with p=0.0198.

c. Representative H&E stained and Ki67 IHC stained sections of lungs and pancreas from neonates described in (B). Below: Quantification of Ki67 IHC in neonatal lung (left) and pancreas right) of mice hypomorphed post 13.5 *dpc*. Results represent mean ± SD. The unpaired t-test with Welch's correction and two-tailed analysis was used to analyze data. For lung, n=4 for both non-hypomorphed control and hypomorphed mice with p=0.8080. For pancreas, n=3 for both non-hypomorphed control and hypomorphed mice with p=0.6275.

*p < 0.05, ns = non-significant. SD = standard deviation.

Source data are provided as a Source Data file.

Sodir *et al.* Supplementary Figure 9:
Imposition of Myc hypomorphism immediately after 13.5 *dpc* has no discernible impact on subsequent development or neonatal growth

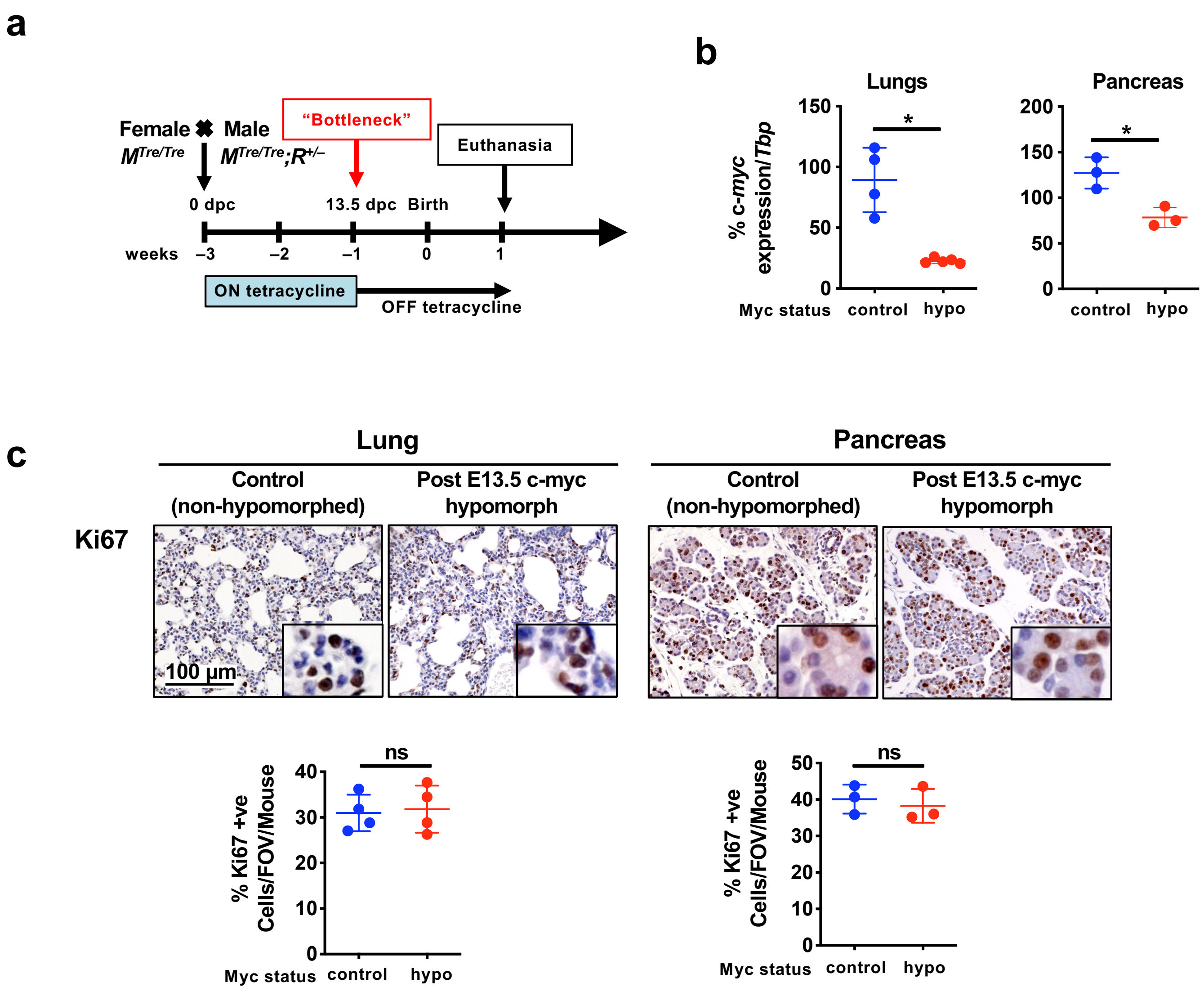

Supplementary Figure 10: Full physiological Myc levels are required to maintain normal haematopoiesis

a. *MR* mice were maintained on continuous tetracycline through development up to 5 weeks post-partum. Tetracycline was then withdrawn to hypomorph Myc and peripheral blood serially collected every 6 weeks thereafter (red). Control mice were maintained on tetracycline throughout (blue). The numbers of leukocytes (left), erythrocytes (middle), and platelet (right) are expressed relative to age-matched tetracycline treated controls. Results depict mean + SD. Multiple Unpaired t test with Welch correction, single pooled variance, Holm-Šídák method was used to analyze data. For leukocytes, n=8 and 6 at 0 weeks (adjusted p=0.683077883883092), n=10 and 10 at 6 weeks (adjusted p=0.035710789563789), n=12 and n=11 at 12 weeks (adjusted p=0.009476579685324), n=19 and 11 at 18 weeks (adjusted p=0.000001067152537), n=12 and 7 at 24 weeks (adjusted p=0.000001082880949), and n=10 and 8 at 30 weeks (adjusted p=0.000002031607466), for non-Myc hypomorphed and Myc hypomorphed mice, respectively. For erythrocytes, n=7 and 7 at 0 weeks (adjusted p=0.678004234441236), n=10 and 10 at 6 weeks (adjusted p=0.189296851840331), n=12 and 11 at 12 weeks (adjusted p=0.000000000201341), n=15 and 7 at 18 weeks (adjusted p=0.000000000015804), n=13 and 8 at 24 weeks (adjusted p=0.000000000001262), and n=3 and 5 at 30 weeks (adjusted p=0.000009806897331), for non-Myc hypomorphed and Myc hypomorphed mice, respectively. For Platelets, n= 8 and 7 at 0 weeks (adjusted p=0.948394035639912), n=10 and 10 at 6 weeks (adjusted p=0.914488378388986), n=12 and 5 at 12 weeks (adjusted p=0.948394035639912), n=7 and 7 at 18 weeks (adjusted p=0.000004535104808), n=8 and 8 at 24 weeks (adjusted p=0.000006748654704), and n=5 and 7 at 30 weeks (adjusted p=0.000039162665868), for non-Myc hypomorphed and Myc hypomorphed mice, respectively

*p < 0.05, **p < 0.01, ***p < 0.001, ****p < 0.0001. SD = standard deviation.

b. Representative H&E-stained sections of spleen from control *MR* mice (maintained on tetracycline) versus 15 weeks following tetracycline withdrawal.

Source data are provided as a Source Data file.

Sodir *et al.* Supplementary Figure 10:
Full physiological Myc levels are required to maintain normal haematopoiesis

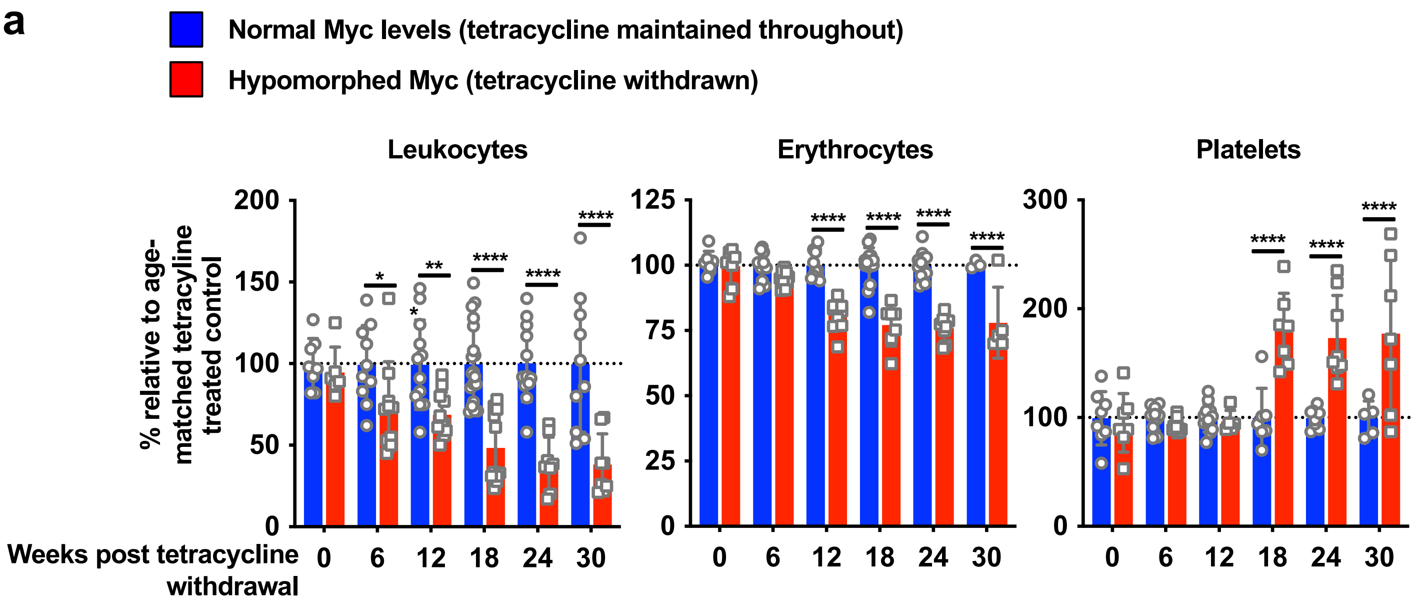

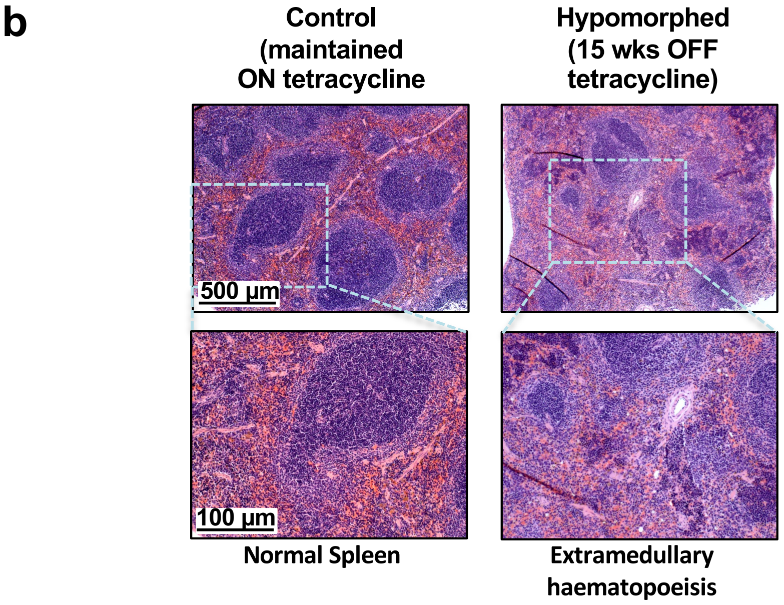

Supplement: Supplementary file 1 — Supplementary Information [file 41467_2022_34079_MOESM1_ESM.pdf]
