# Supplementary material for: Reversible Myc hypomorphism identifies a key Myc-dependency in early cancer evolution
Source: Nat Commun. 2022 Nov 9;13:6782. doi: 10.1038/s41467-022-34079-x (PMC9646778; doi:10.1038/s41467-022-34079-x)
Supplement: Supplementary file 3 — Reporting Summary [file 41467_2022_34079_MOESM3_ESM.pdf]

nature research

Corresponding author(s): Gerard I Evan and Nicole M Sodir

Last updated by author(s): 2022/09/08

Reporting Summary

Nature Research wishes to improve the reproducibility of the work that we publish. This form provides structure for consistency and transparency in reporting. For further information on Nature Research policies, see our Editorial Policies and the Editorial Policy Checklist.

Statistics

For all statistical analyses, confirm that the following items are present in the figure legend, table legend, main text, or Methods section.

n/a	Confirmed	
☐	☒	The exact sample size (n) for each experimental group/condition, given as a discrete number and unit of measurement
☐	☒	A statement on whether measurements were taken from distinct samples or whether the same sample was measured repeatedly
☐	☒	The statistical test(s) used AND whether they are one- or two-sided<br>*Only common tests should be described solely by name; describe more complex techniques in the Methods section.*
☒	☐	A description of all covariates tested
☒	☐	A description of any assumptions or corrections, such as tests of normality and adjustment for multiple comparisons
☐	☒	A full description of the statistical parameters including central tendency (e.g. means) or other basic estimates (e.g. regression coefficient) AND variation (e.g. standard deviation) or associated estimates of uncertainty (e.g. confidence intervals)
☐	☒	For null hypothesis testing, the test statistic (e.g. F, t, r) with confidence intervals, effect sizes, degrees of freedom and P value noted<br>*Give P values as exact values whenever suitable.*
☒	☐	For Bayesian analysis, information on the choice of priors and Markov chain Monte Carlo settings
☒	☐	For hierarchical and complex designs, identification of the appropriate level for tests and full reporting of outcomes
☒	☐	Estimates of effect sizes (e.g. Cohen's d, Pearson's r), indicating how they were calculated

Our web collection on statistics for biologists contains articles on many of the points above.

Software and code

Policy information about availability of computer code

Data collection	Microscope images where acquired with Zeiss Axiovision Rel 4.8 software. Quantitative PCR data were collected with Eppendorf Quantstudio design & Statistical analysis V1.2 Software. For quantification of tumour burden, H&E sections were scanned with an Aperio AT2 microscope (Leica Biosystems) at 20X magnification (resolution 0.5 microns per pixel) and analyzed with Aperio Software v12.1.0.502. The RNA sequencing data were quality checked using FastQC v0.11.4; reads were trimmed using TrimGalore v0.5.0; reads were mapped using STAR v2.7.1; the Ensembl Mus Musculus GRCm39 (release 103) reference genome was used with annotated transcripts from the Ensembl Mus_Musculus. GRCm39.103.gtf file; and the number of reads mapping to genomic features was calculated using HTSeq v0.6.1.
Data analysis	Graphs and statistic were performed with GraphPad-Prism version 8 software. For the RNA sequencing data, differential Gene Expression Analysis using the counted reads employed the R package edgeR v3.26.5 and the data were bias corrected using the CQN package (version 1.24.0).

For manuscripts utilizing custom algorithms or software that are central to the research but not yet described in published literature, software must be made available to editors and reviewers. We strongly encourage code deposition in a community repository (e.g. GitHub). See the Nature Research guidelines for submitting code & software for further information.

Data

Policy information about availability of data

All manuscripts must include a data availability statement. This statement should provide the following information, where applicable:

- Accession codes, unique identifiers, or web links for publicly available datasets
- A list of figures that have associated raw data
- A description of any restrictions on data availability

All data generated or analysed during this study are included in this published article (and its supplementary information files).In addition, the raw data are available

in the Source Data file whenever possible. Source data are provided with this paper. The RNA-seq data generated and analyzed in this study have been deposited in the ArrayExpress database at EMBL-EBI (www.ebi.ac.uk/arrayexpress) under accession number E-MTAB-10807 (https://www.ebi.ac.uk/arrayexpress/experiments/E-MTAB-10807/).

Field-specific reporting

Please select the one below that is the best fit for your research. If you are not sure, read the appropriate sections before making your selection.

- [x] Life sciences
- [ ] Behavioural & social sciences
- [ ] Ecological, evolutionary & environmental sciences

For a reference copy of the document with all sections, see nature.com/documents/nr-reporting-summary-flat.pdf

Life sciences study design

All studies must disclose on these points even when the disclosure is negative.

Sample size	Sample size was determined on calculations based on our previous published data (https://doi.org/10.1158/2159-8290.CD-19-0435, https://doi.org/10.1016/j.cell.2017.11.013), showing that a sample size of n = 5 to 6 mice per group allows detection of the (large) effect sizes of interest with a probability greater than 0.9. Advice for all studies was sought from our biostatistician.
Data exclusions	No data were excluded from the analysis
Replication	Biological (the number of mice was equal to or above 3 mice) and technical replicates (RT-PCR data were run in triplicates) were used. All attempts at replication were successful.
Randomization	Mice were age-matched and their allocation was random ; however we made sure that each group includes both sexes (females and males)
Blinding	Investigators were blinded to group allocation during data collection and analysis

Reporting for specific materials, systems and methods

We require information from authors about some types of materials, experimental systems and methods used in many studies. Here, indicate whether each material, system or method listed is relevant to your study. If you are not sure if a list item applies to your research, read the appropriate section before selecting a response.

Materials & experimental systems

n/a	Involved in the study	
[ ]	[x]	Antibodies
[x]	[ ]	Eukaryotic cell lines
[x]	[ ]	Palaeontology and archaeology
[ ]	[x]	Animals and other organisms
[x]	[ ]	Human research participants
[x]	[ ]	Clinical data
[x]	[ ]	Dual use research of concern

Methods

n/a	Involved in the study	
[x]	[ ]	ChIP-seq
[x]	[ ]	Flow cytometry
[x]	[ ]	MRI-based neuroimaging

Antibodies

Antibodies used	Rabbit monoclonal anti-Ki67 (clone SP6, 1:100 dilution)) (Lab Vision, Fisher Scientific, RM9106S1) lot# 910651901K , goat polyclonal anti-CD206 antibody (1:200 dilution, R&D Systems, AF2535) lot# WFT0320011, rabbit monoclonal IL-23 (EPR5585(N), 1:100 dilution) (Abcam, ab190356) lot# GR3241587-5, rabbit monoclonal anti-CD3 (Clone SP7, undiluted) (Fisher Scientific, RM9107RQ), mouse monoclonal anti-NKp46 (clone 29A1.4, 1:100 dilution) (Biolegend, 137601) lot# B267691, rabbit monoclonal anti-Myc (Y69, 1:500 dilution for IHC and 1:2,000 dilution for western blot) (Abcam, ab32072) lot # GR189790-33, and β-actin (sc-69879, Santa-Cruz Biotechnology, 1:5,000 dilution) lot# G3120, HRP-conjugated secondary antibodies: goat anti-rabbit (sc-2301; Santa-Cruz Biotechnology 1:7,500 dilution) and goat anti-mouse (A4416, Sigma-Aldrich, 1:7,500 dilution).
Validation	Antibodies used in our study are all commercially available; they were validated by the manufacturer (advanced validation) and on our positive control samples.<br>Anti-Ki67 ab validated on spleen, tonsil and breast cancer (https://www.fishersci.com/shop/products/ki-67-rabbit-monoclonal-antibody/RM9106S1); anti-CD206 validated on mouse testis and lungs (https://www.rndsystems.com/products/mouse-mmr-cd206-antibody_af2535); anti-IL-23 validated on THP-1 treated with GM-CSF+IL-4, THP-1 and Raji cell lysates; IL23 recombinant protein (Human IL23 p40 (aa23 -aa328) & Human IL23 P19 (aa21-aa189) (65KDa) (https://www.abcam.com/il-23-antibody-epr5585n-c-terminal-ab190356.html); anti-CD3 validated on Jurkat cells, Tonsil (https://www.fishersci.com/shop/products/lab-vision-cd3-early-t-cell-marker-rabbit-monoclonal-antibody/RM9107RQ); anti-NKp46 validated on C57BL/6 mouse splenocytes (https://www.biolegend.com/nl-be/products/purified-anti-mouse-cd335-nkp46-antibody-6526); anti-Myc validated on Burkitt lymphoma with a confirmed c-Myc translocation (https://www.abcam.com/c-myc-antibody-y69-chip-grade-ab32072.html); β-actin validated by western blot on A-10 and 293T cells (https://www.scbt.com/p/beta-actin-antibody-ac-15); HRP-conjugated secondary antibodies:

goat anti-rabbit validated by western blot of MEK-2 expression in HeLa whole cell lysate (https://datasheets.scbt.com/sc-2301.pdf); and goat anti-mouse validated on https://www.sigmaaldrich.com/US/en/product/sigma/a4416.

Animals and other organisms

Policy information about studies involving animals; ARRIVE guidelines recommended for reporting animal research

Laboratory animals	p53flox; LSL-KrasG12D, Pdx-1-Cre, LSL-p53R172H, Rosa26-LSL-MERT2 and MycTRE and actin-tTS mice were used in this study. They are mixed strain but backcrossed to C57BL/6 background. Mice enrolled in a specific experiment were age-matched and include both sexes (females and males). Depending on the experimental approach, mice were enrolled between 4 to 8 weeks of age. Mice were maintained on a 12-hour light/dark cycle with continuous access to food and water. The environmental conditions were as follows: temperature 20-24°C (68-75°F) and humidity 55% +/- 10%.
Wild animals	The study did not involve wild animals
Field-collected samples	All mice were inbred laboratory strains. No field collected samples were used in this study.
Ethics oversight	All animal studies were reviewed and approved by the Animal Welfare and Ethical Review Body (AWERB) of the Cancer Research UK Cambridge Institute and the Francis Crick Institute and licensed by the Home Office (license number PP2645677 and protocols numbers 2 and 3). The ARRIVE guidelines were adhered to throughout.

Note that full information on the approval of the study protocol must also be provided in the manuscript.
